# An explorative study of metabolic responses to mental stress and yoga practices in yoga practitioners, non-yoga practitioners and individuals with metabolic syndrome

**DOI:** 10.1186/1472-6882-14-445

**Published:** 2014-11-15

**Authors:** Anupama Tyagi, Marc Cohen, John Reece, Shirley Telles

**Affiliations:** RMIT, West Campus, Building 201, Level 4, Bundoora, Vic 3083 Australia; PO Box 71, Bundoora, Vic 3083 Australia; Patanjali Research Foundation, Bahdrabad, Haridwar, Uttrakhand 249402 India

**Keywords:** Yoga, Meditation, Breathing, Metabolic syndrome, Oxygen consumption, Energy Expenditure, Metabolic rate, Stress reactivity, Stress recovery

## Abstract

**Background:**

Stress places a metabolic burden on homeostasis and is linked to heightened sympathetic activity, increased energy expenditure and pathology. The yogic state is a hypometabolic state that corresponds with mind-body coherence and reduced stress. This study aimed to investigate metabolic responses to stress and different yoga practices in regular yoga practitioners (YP), non-yoga practitioners (NY) and metabolic syndrome patients (MS).

**Methods:**

YP (n = 16), NY (n = 15) and MS (n = 15) subjects underwent an experimental protocol that comprised of different 5-minute interventions including mental arithmetic stress test (MAST), alternate nostril breathing (ANB), *Kapabhati* breathing (KB) and meditation (Med) interspersed with 5 minutes of quiet resting (neutral condition (NC)). During the intervention periods continuous body weight adjusted oxygen consumption (VO2ml/min/kg) was measured using open circuit indirect calorimetry with a canopy hood.

**Results:**

This is the first study to report oxygen consumption (OC) in yoga practitioners during and after MAST and the first to report both within and between different populations. The results were analysed with SPSS 16 using 3X9 mixed factorial ANOVAs. The single between-subject factor was group (YP, NY and MS), the single within-subject factor was made up of the nine intervention phases (NC1, MAST, NC2, ANB, NC3, KB, NC4, Med, NC5). The results demonstrated that the regular YP group had significantly less OC and greater variability in their OC across all phases compared to the MS group (*p* = .003) and NY group (*p* = .01). All groups significantly raised their OC during the mental arithmetic stress, however the MS group had a significantly blunted post-stress recovery whereas the YP group rapidly recovered back to baseline levels with post stress recovery being greater than either the NY group or MS group.

**Conclusions:**

Yoga practitioners have greater metabolic variability compared to non-yoga practitioners and metabolic syndrome patients with reduced oxygen requirements during resting conditions and more rapid post-stress recovery. OC in metabolic syndrome patients displays significantly blunted post-stress recovery demonstrating reduced metabolic resilience. Our results support the findings of previous randomised trials that suggest regular yoga practice may mitigate against the effects of metabolic syndrome.

**Clinical trial number:**

ACTRN12614001075673; Date of Registration: 07/10/2014.

**Electronic supplementary material:**

The online version of this article (doi:10.1186/1472-6882-14-445) contains supplementary material, which is available to authorized users.

## Background

Stress has been defined as a ‘nonspecific response of the body to any noxious stimulus’[1] and the stress response is associated with heightened sympathetic nervous activity and increased energy expenditure along with associated changes in heart rate, breath rate and blood pressure. Stress places a metabolic burden on homeostatic processes and if stress is severe or prolonged, it may lead to disturbed homeostasis, distress and psychophysiological dysfunction[2] with increased resting metabolic rate[3], exacerbation of metabolic dysfunction[4, 5] and acceleration of aging[6] morbidity and mortality[7]. Several longitudinal studies further suggest that severe prolonged stress is associated with the development of metabolic syndrome[4, 8], which is related to impaired mitochondrial functioning and metabolic inflexibility[9].

Metabolic activity, which is maximal during intense physical activity and lowest during resting conditions, increases with both psychological and physiological activity as well as with stress and many pathological conditions[7, 10]. Higher oxygen consumption (OC) is seen with mental arithmetic or video gaming[11–13] and OC is reported to be higher in people with pathological conditions such as chronic obstructive pulmonary diseases[14], congestive heart failure[15], insomnia[16], anxiety[17], HIV-AIDS[18, 19] as well as the individual features of metabolic syndrome such as hypertension[20, 21], obesity[22, 23], diabetes[24, 25] and dyslipidaemia[26]. Peak oxygen consumption has also been found to be lower in people with metabolic syndrome[19, 27].

While stress increases metabolic activity and dissociates physiological and psychological processes, relaxation practices tend to induce mind-body coherence or a sense of psychological and physiological equilibrium that counteracts stress by inducing a ‘relaxation response’[28]. Yoga includes a range of mind-body practices that include postures, breathing, meditation and relaxation[29], and studies suggest that a single yoga session can lead to improvements in cognitive performance[30], baroreflex sensitivity, oxygen saturation[31–33], sympathovagal balance (HRV)[34] and enhance recovery after stressful stimuli[35, 36]. Further studies suggest that regular yoga practice can effectively mitigate workplace stress[37], examination stress[38, 39], stress-induced inflammation[40], caregiver stress[41] and post-traumatic stress[42].

The stress relieving properties of yoga are associated with induction of a hypo-metabolic or ‘yogic state’ state that is associated with reduced psychophysiological activity and OC[43], with reduction in OC of up to 40% during specific practices[44]. This ‘yogic state’, which corresponds to a state of mind-body coherence, has also been described as the ‘flow state’[45] and is distinct from rest[46], physical relaxation[47] and sleep[48] and may be voluntarily induced while performing fixed physiological workloads[49]. The long term practice of yoga may also enhance metabolic resilience and flexibility. Compared to non-yoga practitioners, regular yoga practitioners are reported to have reduced heart[50], breath, and metabolic rates[51, 52] as well as reduced blood pressure[50, 53] and lipid profiles[54]. Regular yoga practice has also been associated with improved lung function[53, 55] and heart rate variability[56] with the duration of practice directly corresponding to reductions in heart rate (HR), blood pressure (BP) and respiratory rate (RR)[54] and improvements in mindfulness[57] and lipid profiles[54].

While there appears to be a clear relationship between yoga and metabolism, no studies have compared the metabolic response to mental stress in yoga practitioners and non-yoga practitioners or compared OC in yoga practitioners, non-yoga practitioners and metabolic syndrome patients during different yoga practices. To fill this gap, the following explorative study was designed to examine the metabolic responses to stress and different yoga practices and compare the responses of regular yoga practitioners, non-yoga practitioners and metabolic syndrome patients.

## Methods

### Study location

The study took place between November and January 2012 at the Yoga Research laboratory situated at *Patanjali Yogpeeth* in *Haridwar*, India. The study was approved by the RMIT University Human Research Ethics Committee and retrospective approval was granted by the ethics committee at *Patanjali Yogapeeth*.

### Population

The study population involved three groups aged 18 years to 55 years all of Indian origin. The groups were:

### Yoga practitioner (YP) Group

Consisted of yoga teachers, yoga therapists and yoga active persons with a minimum of 6 months yoga experience who were residents of the *Patanjali Yogpeeth* campus or surrounding *ashrams* and were involved in routine ashram life. They practiced yoga for at least 90 minutes daily and were non-smokers on a strict non-alcoholic, vegetarian diet for at least 6 months.

### Non-Yoga practitioner (NY) Group

Included temporary residents of *Patanjali Yogpeeth* and at the time of their participation were all non-smokers on a non-alcoholic, vegetarian diet. Most of the participants in this group had never practiced yoga before, but some had either practiced irregular or recently begun yoga practice (5 days).

### Metabolic Syndrome (MS) Group

Included subjects with metabolic syndrome as defined by the International Diabetic Federation[58] and diagnosed by their general practitioner and treated with regular medication or prescribed lifestyle modification. These subjects, who were not residents of the campus and had not previously practiced yoga were asked to maintain a vegetarian diet and refrain from tobacco and alcohol 10 hours prior to their experimental session.

People with any serious medical condition or condition requiring regular analgesic medication, or who were unable to adequately perform the yogic interventions were excluded. Pregnant women and women in their menstrual period were also excluded to avoid the cyclic decrease in basal metabolic rate prior to ovulation[59].

### Experimental protocol

All participants were requested to have of minimum 7 hours sleep and avoid strenuous exercise, alcohol and analgesic medication the day before their experimental session. Participants were asked to attend wearing loose comfortable clothing and to fast and abstain from tobacco and caffeine for 8 hours prior to their session. All the sessions for the YP group were conducted between 7 to 9 AM while the sessions for the NY and MS groups took place between 8 to 10 AM except for 5 NY participants who were unavailable in the mornings and had sessions conducted during the afternoon.

On attending the laboratory, participants had the study explained to them in their native language by the experimenter and detailed information with demonstration of the each intervention was provided. The YP were all well versed with the specific yogic interventions. Some of the NY had brief acquaintance with some practices, while none of the MS group had prior experience with yoga and were given time to practice and get acquainted with the interventions till they performed them accurately. As Hindi was the native language of most participants, the consent forms and demographic forms were all translated into Hindi language by professional translators. Reliability and validity of the translation was checked via a different group of professional translators providing back translation to English.

After providing written consent, participants were asked to empty their bladder and switch off their mobile phones. They then had their height, body weight, waist circumference, seated BP and radial artery pulse measured before commencing the experimental session. All measurements were performed by the same experimenter with participants in a recumbent position. The laboratory was maintained at a comfortable temperature of 23 degrees Celsius with subdued lighting.

**Figure 1:**
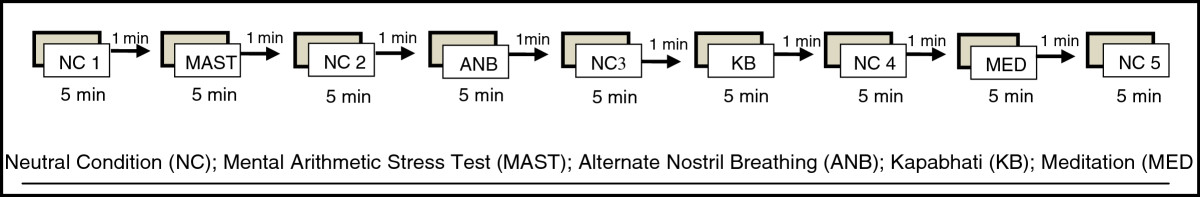
**Experimental sequences.**

Before gas exchange recording commenced, subjects were asked to relax and adjust to the environment of the canopy for 10 minutes. The experimental session lasted around 60 minutes and involved multiple phases of equal duration with intervention phases interspersed with the neutral condition of eyes open rest. The experimental sequence involved nine separate 5 minute phases, each preceded by a one minute pause **(Pause)** during which BP was measured and they were instructed on the next phase (Figure 1). The yogic practices were all based on traditional yoga texts with some slight variation to account for the recumbent position and the requirements of the measuring equipment.

### Neutral Condition (NC)

During the neutral condition participants were instructed to breathe spontaneously, avoid movement, yawning or mental agitation and have a relaxed quiet mind.

### Mental Arithmetic Stress Test (MAST)

The Mental Arithmetic Stress Test (MAST) involved participants having their eyes closed and being instructed in a crisp tone of voice to read a 4 digit number written on a piece of cardboard and to then count backwards by 3 s as quickly and accurately as possible until asked to stop.

### Alternate Nostril Breathing (ANB)

The ANB phase involved subjects gently occluding alternate nostrils with their fingers as described by Niranjanananda[60] and continue this cycle rhythmically, soundlessly and effortlessly for 5 minutes until instructed to stop. The experienced yoga practitioners were informed not to perform retention during breathing cycles.

### Kapalbhati Breathing (KB)

Participants were instructed to breathe through their nostrils at around 48 BPM (0.8 Hz) and forcefully exhale by contracting their abdominal muscles and then allowing inhalation to occur spontaneously. As this type of yogic breathing can be stressful and result in dizziness or discomfort for some people, the non-yoga and MS group were suggested to continue a cycle of spontaneous breathing in the event of any discomfort.

### Meditation (Med)

The meditation phase involved subjects lying with their body relaxed and still and eyes gently closed. Subjects were asked to quietly repeat the mantra ‘AUM’ at their own pace during exhalation.

### Outcome measures

#### Anthropometric measures

Body weight and height were measured using an electronic platform scale (Gold Tec GTEP-100 K), and stadiometer (Seca-274). Waist circumference was measured with subjects standing with arms at their side, feet close together and weight evenly distributed. Measures were taken at the midpoint between the lower margin of the last palpate rib and the top of the iliac crest at the end of expiration to ensure a relaxed abdominal wall as per WHO guidelines[61].

#### BP measurement

Blood pressure was measured before and after every experimental condition using an automatic digital blood pressure monitor (Welch Allyn, Redding Medical, USA) with the cuff positioned on the left arm at the level of the right atrium. The BP monitor was calibrated and checked using a mercury sphygmomanometer and stethoscope as described by the British Hypertension Society[62].

#### Metabolic measures

This study focused on weight-adjusted OC (relative OC) which is more reliable when comparing people with different body weight[63].

**VO2ml/min/kg (Relative OC)** was measured continuously via indirect calorimeter using an open circuit, OC gas analyser. This calorimeter uses a canopy hood and dilution technique that allows measurements to be made with subjects breathing spontaneously or as instructed according to the study protocol without the encumbrance of a face mask, mouth piece or nose clips (Quark CPET, Italy). Before each experimental session the calorimeter was calibrated using a reference gas mixture.

Measurement of O2 consumption and CO2 production was performed at 5 second intervals. Calibration of flow was performed using a certified 3 L calibrated syringe and calibration of the O2 and CO2 gas analysers was performed prior to each experimental session using a certified calibration gas. Before each experimental session a 5 minute steady state was achieved as per previous studies[64]. The calorimeter provided both absolute and relative (per kg of body weight) values.

## Results

Demographic details of the Participants (Table 1).

**Table 1:** **Demographic properties of the participants**

Group	Gender	Age (yrs)	Weight (kg)	BMI
Yoga practitioners (YP) (n = 16)	F = 3	32 ± 10	62 ± 7	21.1 ± 1.6
	M = 12			
Non-Yoga practitioners (NY) (n = 15)	F = 5	30 ± 11	69 ± 12	24.9 ± 4.0
	M = 10			
Metabolic syndrome (MS) (n = 15)	F = 3	41 ± 5	86 ± 9	31.5 ± 1.7
	M = 12			

### Statistical analysis

Data were analysed using SPSS 19. Metabolic variable- relative OC (VO2ml/min/kg) was analysed using a series of 3 × 9 mixed factorial ANOVAs. The single between-subjects factor was group (Yoga Practitioner, Non-yoga Practitioners and Metabolic Syndrome); the single within-subjects factor was made up of the nine levels of Phase (NC1, MAST, NC2, ANB, NC3, KB, NC4, MED, NC5). Any significant group by phase interactions were followed by a full analysis of simple main effects. The descriptive values of metabolic variable across nine phases in three groups are shown in Table 2.

**Table 2:** **Means and standard deviations of VO2ml/min/kg of all three groups across nine phases**

	Phases								
Groups	NC1	MAST	NC2	ANB	NC3	KB	NC4	Med	NC5
**YP**	3.6 ± 0.2	4.1 ± 0.2	3.7 ± 0.2	4.4 ± 0.2	3.6 ± 0.3	4.5 ± 0.2	3.8 ± 0.1	3.5 ± 0.1	3.6 ± 0.2
**NY**	3.8 ± 0.2	4.3 ± 0.2	4.0 ± 0.2	4.4 ± 0.2	3.9 ± 0.2	4.4 ± 0.2	4.0 ± 0.2	3.8 ± 0.2	3.8 ± 0.2
**MS**	3. 9 ± 0.2	4.2 ± 0.3	4.2 ± 0.2	4.1 ± 0.3	3.9 ± 0.2	4.3 ± 0.2	4.2 ± 0.2	3.9 ± 0.3	3.9 ± 0.3

**Group:** YP = Yoga Practitioners; NY = Non- Yoga Practitioners; MS = Metabolic Syndrome Patients.

**Phases:** NC = Neutral Condition; MAST = Mental Arithmetic Stress Test; ANB = Alternate Nostril Breathing; KB = Kapalbhati Breathing; Med = Meditation.

#### Inferential results for metabolic variable (VO2ml/min/kg)

**Figure 2:**
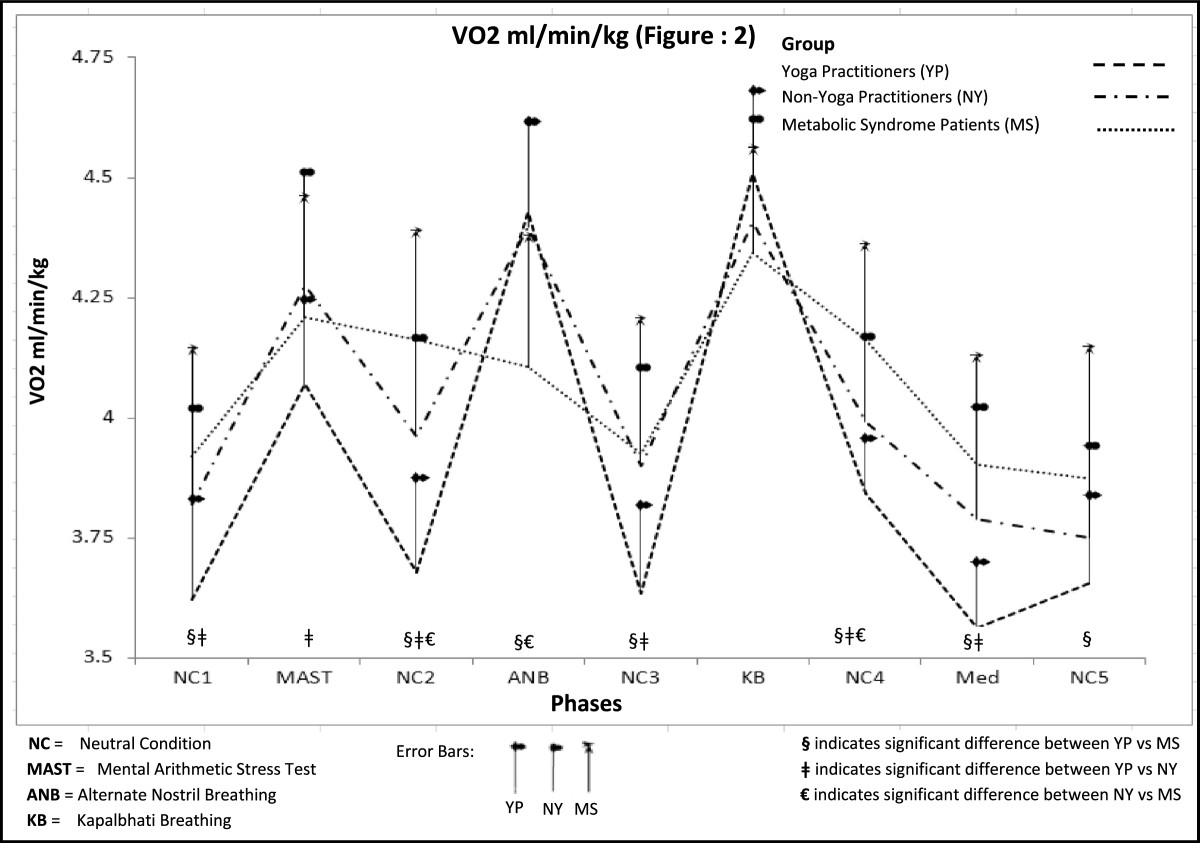
**Pattern of change in VO2ml/min/Kg for 3 groups across the nine experimental phases.**

Figure 2 shows the pattern of changes in relative oxygen consumption (VO2/min/kg) for 3 groups over the nine experimental phases.

Statistical analysis revealed a significant phase by group interaction, Λ = .11, *F*(16, 72) =8.66, *p* < .001, η_p_^2^ = .66, a significant phase main effect, Λ = .04, *F*(8, 36) =122.25, *p* < .001, ηp 2 = .96, along with significant group main effect, Group, *F* (2, 43) = 5.85, *p* = 006, η_p_^2^ = .21. The pairwise comparison of the estimated marginal means for the main effect for group revealed a significant difference in relative OC between the YP group and both MS and NY groups, and has been illustrated in Table 3).

**Table 3:** ***P*** **values for pairwise comparison of estimated marginal means of VO2ml/min/Kg by group**

All phase	YP vs MS	YP vs NY	NY vs MS
Estimated marginal means	.003*	.01*	.15

YP = Yoga practitioners; NY = Non-Yoga Practitioners; MS = Metabolic Syndrome Patients.

* = p value significant.

Table 4 illustrates, p values for pairwise mean comparisons based on the analysis of estimated marginal means with Bonferroni-adjusted α values revealed significant differences between the YP and MS groups in phases NC1, NC2, ANB, NC3, NC4, Med and NC5. Significant differences were also found between the YP and NY groups in phases NC1, MA, NC2, NC3, NC4 and Med. The NY group was significantly different from the MS group at NC2, ANB and NC4.

**Table 4:** ***P*** **values for pairwise comparison based of estimated marginal means with Bonferroni adjusted α values**

Phases	YP vs MS	YP vs NY	NY vs MS
NC 1	001*	.03*	NS
MAST	NS	.04*	NS
NC 2	<.001*	.008*	.01*
ANB	.001*	NS	.003*
NC 3	.003*	.006*	NS
KB	NS	NS	NS
NC 4	<.001*	.04*	.05*
Med	.001*	.01*	NS
NC 5	.02*	NS	NS

**Group**: YP = Yoga Practitioner; NY = Non-Yoga Practitioner; MS = Metabolic Syndrome Patients.

**Phases**: NC = Neutral Condition; MAST = Mental Arithmetic Stress Test; ANB- Alternate Nostril Breathing; KB = Kapalbhati Breathing; Med = Meditation. NS = Not Significant.

* = p values significant.

Analysis of the simple main effect for phase within group revealed that the three groups differed significantly in every phase except for during *Kapalbhati* breathing: phase 1- ‘Neutral Condition 1′ (NC 1), *F*(2, 43) =7.97, *p* = .001, η_p_^2^ = .27; phase 2 ‘Mental Arithmetic Stress Test ’ (MAST), *F*(2, 43) =3.46, *p* = .04, η_p_^2^ = .14; Phase 3- ‘Neutral Condition 2′ (NC 2), *F*(2, 43) =19.34, *p* < .001, η_p_^2^ = .47; Phase 4- ‘Alternate Nostril Breathing’ (ANB), *F*(2, 43) =9.14, *p* < .001, η_p_^2^ = .29; Phase 5- ‘Neutral Condition 3′ (NC 3), *F*(2, 43) =7.91, *p* = .001, η_p_^2^ = .27, Phase 7- ‘Neutral condition 4′ (NC 4), *F*(2, 43) =14.34, *p* < .001, η_p_^2^ = .40; Phase 8- Meditation (Med), *F*(2, 43) =11.36, *p* < .001, η_p_^2^ = .35 and Phase 9- ‘Neutral Condition 5′ (NC 5) *F*(2, 43) =3.82, *p* = .03, η_p_^2^ = .15.

Further, simple main effect analysis for group within phase revealed that all three groups varied significantly across the nine phases: Yoga Practitioners (YP), Λ = .05, *F*(8, 36) =88.32, *p* < .001, η_p_^2^ = .95; Non-yoga Practitioners (NY), Λ = .10, *F*(8, 36) =41.47, *p* < .001, η_p_^2^ = .89 and Metabolic Syndrome (MS), Λ = .19, *F*(8, 36) =18.54, *p* < .001, η_p_^2^ = .80. The YP group demonstrated the largest variability across the nine phases, as evidenced by the higher effect size value of η_p_^2^_=_ .95 for the yoga group in the group within phase simple main effect, by comparisons with a figure of η_p_^2^ = .90 for the NY group and .80 for the MS group. This effect size value is derived from the corresponding *F* ratios, which also demonstrated considerably more variance in the YP group *F* (8, 36) =82.86, *p* < .001, compared with both NY group *F* (8, 36) =39.68, *p* < .001 and the MS group *F* (8, 36) =18.24, *p* < .001. Across all phases, the yoga practitioner group was significantly different from both the NY group, *p* < .017 and the MS group, *p* < .001.

At the level of pairwise comparison for simple main effect of groups within phases, several significant differences were evident in the three groups in different phases (Table 5 describes: *p* values of comparison of each intervention with pre and post phase).

**Table 5:** ***P*** **values for comparison of each intervention with pre and post phase**

Phases	Yoga practitioner	Non-Yoga practitioner	Metabolic syndrome patients
NC1 vs MAST	<.001*	.001*	.001*
MAST vs NC2	<.001*	.009*	NS
NC2 vs ANB	<.001*	.001*	NS
ANB vs NC3	<.001*	.007*	NS
NC3 vs KB	< .001*	< .001*	< .001*
KB vs NC4	< .001*	.006*	.003*
NC4 vs MED	< .001*	.002*	.008*
MED vs NC5	NS	NS	NS

**Phases:** NC = Neutral condition; MAST = Mental Arithmetic Stress Test; ANB = Alternate Nostril Breathing; KB = Kapalbhati Breathing; Med = Meditation;

NS = Not Significant; * = p values significant.

There is also some evidence indicating that YP group exhibited a higher level of recovery that the other two groups. The effect sized of each group associated with the change from phase 2 to phase 3 revealed a value of *d* = 0.37 for the MS group, *d* = 2.05 for the NY group and *d* = 1.98 for the YP group. The changes from phase 2 to 3 was not significant for MS group but was significant at *p* = .009 and *p* < .001 respectively for NY and YP groups.

## Discussion

This observational study found that regular yoga practitioners had significantly less OC and greater variability in their OC across all phases compared to non-yoga practitioners or metabolic syndrome patients. While the YP group had lower baseline OC, their OC increased substantially in response to the active interventions and fully recovered back to baseline levels during resting periods. These findings related to metabolic activity are consistent with previous studies that have found regular yoga practitioners have a lower basal metabolic rate compared to non-yoga practitioners[51, 52] with greater recovery from stress[65, 66].

This is the first study to report OC in yoga practitioners during and after mental arithmetic stress and the first to measure metabolic reactivity and recovery both within and between different participant populations involving metabolic syndrome patients. Our study is also the first to examine OC in yoga practitioners using indirect calorimetry with a ventilatory hood. While other studies have examined yoga using similar indirect calorimetry methods[67], the use of a ventilatory hood is reported to less stressful than calorimetry that uses a mouth piece or mask[68, 69] and hence is likely to have facilitated greater relaxation amongst our participants and therefore more accurate results.

While our study used a rigorous experimental method, it is an observational study rather than a randomised clinical trial and the differential effects between groups may have been due to factors other than their history of yoga practice. The selection of our study population and protocol may have also introduced some bias. For example there were numerous differences between the groups with the MS group being on average 10 years older than the other groups and the baseline OC being lowest in the YP and highest in the MS groups. Furthermore, there were some smokers in the MS group and none in the YP group and the YP group were all well versed with the yoga practices and were also involved in routine ashram life, which included activities such as meditation, yoga philosophy discussions, meetings with spiritual leaders and teachers, Ayurvedic diet and structured daily routines. Some of the YP group were also familiar with the laboratory testing, whereas the NY and MS groups were not well versed with the yoga techniques or the laboratory and were only temporary ashram residents or resided outside the ashram where they were subjected to the stresses of everyday life. This may have led to the NY and MS groups being less relaxed than the YP group and less able to perform the active practices correctly. Some aspect of the yoga interventions also lacked uniformity, for example there was a difference in the breath rate between groups during *Kapabhati* breathing, due to the advanced nature of these practices, which are difficult to learn and perform by novices. Comparing these practices in novice and advanced practitioners may therefore produce unreliable results unless the practices are specifically paced to achieve uniform breath rates across groups. The timing and sequencing of the different experimental phases may have also influenced the results, as they were of relatively small duration with only brief rest periods that may not have allowed full recovery between the phases. Due to these limitations we have limited our discussion to the analysis of the mental arithmetic stress test, which occurred first in the experimental sequence, and have not discussed the responses to the ANB, KB or meditation practices.

It is evident that mental arithmetic induced a metabolic burden on our participants, with all groups having significantly raised OC during the mental arithmetic phase. Mental arithmetic stress is widely used to elicit β-adrenergic sympatho-adrenal responses in laboratory conditions[12, 70] and cardiovascular reactivity to mental arithmetic-induced stress is reported to be unrelated to personality type[71], yet more pronounced in subjects with high BP[72]. Cardiovascular reactivity to mental stress is also reported to be an independent risk factor for features of metabolic syndrome including hypertension and insulin resistance[70, 73, 74] as well as for atherosclerosis[75] and future cardiovascular risk[76]. However, cardiovascular reactivity is not always associated with negative health outcomes and may be adaptive and reflect behavioural flexibility, energy mobilisation and effective coping rather than pathology[75] as indicated by reports of a negative association between cardiovascular reactivity and obesity, depression and self-reported health[77].

We found that while mental stress placed a metabolic burden on all groups, the MS group had a significantly blunted post-stress recovery. This supports previous suggestions that recovery responses may be a better predictor of subsequent cardiovascular risk than stress-induced reactivity[78–81]. Our finding that the YP group had a greater post-stress recovery in oxygen consumption than either the NY or MS group are consistent with reports of greater recovery in heart rate in meditators compared to non-meditators after watching a stressful film[65] and greater recovery in self-reported measures after watching negative emotion-evoking slides[66]. Our results further support suggestions that individuals who are able to rapidly return their cardiovascular activity to baseline following a stressful event are more likely to have better cardiovascular health[75].

While the biological mechanism linking stress responses and cardiovascular diseases and mortality is poorly understood, there is evidence to suggest that cardiovascular responses to psychological stress are associated with increased allostatic load[82] and thus poor cardiovascular health[4]. This may be indicated by changes in the autonomic nervous system, hypothalamic-pituitary-adrenal axis and metabolic, immune, cellular and physiological and psychological changes that include endothelial dysfunction, shortened telomere length and less focused thought processes[6, 83–85]. Slower recovery from psychological stress may also reflect a greater allostatic load and reduced sympatho-adrenal flexibility[82], while yoga and meditation appears to enhance adaptability and post-stress recovery[44]. It has been further suggested that the relaxation response may be associated with improved mitochondrial energy production and utilisation that promotes mitochondrial resilience through upregulation of ATPase and insulin function[86].

In our study the YP group had a greater metabolic response to mental arithmetic stress than the other groups as well as having a more rapid recovery. This is consistent with previous studies that suggest that yoga practices enhance autonomic regulatory reflex mechanisms associated with stress[87] and improve autonomic function, pulmonary function and metabolic function[88–90]. Single yoga sessions have also been shown to reduce HR and BP in sedentary individuals[91], healthy non-yoga practitioners[35], as well as in patients with hypertension[92–94] and congestive heart failure[95]. Regular yoga practice has been further shown to down regulate the HPA axis and sympatho-adrenal pathways with reduction in catecholamine and cortisol levels[29, 41], and improve immune response[38, 87], vascular function[96] and melatonin secretion[97, 98]. Regular yoga practice has also been shown to contribute to cognitive improvement[29] physical relaxation[99] and reductions in emotional distress[100] and anxiety[101].

The results of this study suggest that regular yoga practitioners have enhanced metabolic resilience with reduced basal metabolic demands and enhanced recovery after stress while metabolic syndrome patients have higher basal metabolic demands and blunted post-stress recovery. These results are consistent with other studies that report that regular yoga practice enhances metabolic function and improves obesity[102], dyslipidemia[103], hyperglycaemia[104] hypertension[105, 106] and metabolic syndrome[107–109].

## Conclusion

Yoga practitioners have reduced oxygen requirements during resting conditions and greater metabolic flexibility compared to non-yoga practitioners and metabolic syndrome patients. Yoga practitioners are also better able to respond to and recover from the increased metabolic burden due to mental arithmetic stress, while metabolic syndrome patients have significantly blunted post-stress recovery. Further, long term studies are needed in order to establish, if regular yoga practices have an influential role in reducing resting metabolic demand. In future, studies should incorporate longer intervention phases to investigate the metabolic reactivity and recovery to stress and to determine if yoga practices are able to enhance metabolic resilience in metabolic syndrome patients.

## Authors’ original submitted files for images

Below are the links to the authors’ original submitted files for images. Authors’ original file for figure 1 Authors’ original file for figure 2
