# Supplementary material for: An explorative study of metabolic responses to mental stress and yoga practices in yoga practitioners, non-yoga practitioners and individuals with metabolic syndrome
Source: BMC Complement Altern Med. 2014 Nov 15;14:445. doi: 10.1186/1472-6882-14-445 (PMC4247158; doi:10.1186/1472-6882-14-445)
Supplement: Supplementary file 1 — Authors’ original file for figure 1 [file 12906_2013_2013_MOESM1_ESM.pdf]

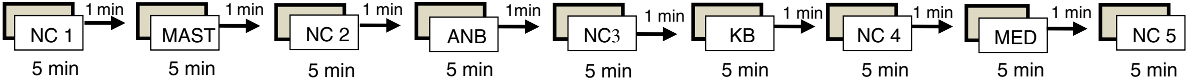

Neutral Condition (NC); Mental Arithmetic Stress Test (MAST); Alternate Nostril Breathing (ANB); Kapabhati (KB); Meditation (MED
