# Supplementary material for: An explorative study of metabolic responses to mental stress and yoga practices in yoga practitioners, non-yoga practitioners and individuals with metabolic syndrome
Source: BMC Complement Altern Med. 2014 Nov 15;14:445. doi: 10.1186/1472-6882-14-445 (PMC4247158; doi:10.1186/1472-6882-14-445)
Supplement: Supplementary file 2 — Authors’ original file for figure 2 [file 12906_2013_2013_MOESM2_ESM.pdf]

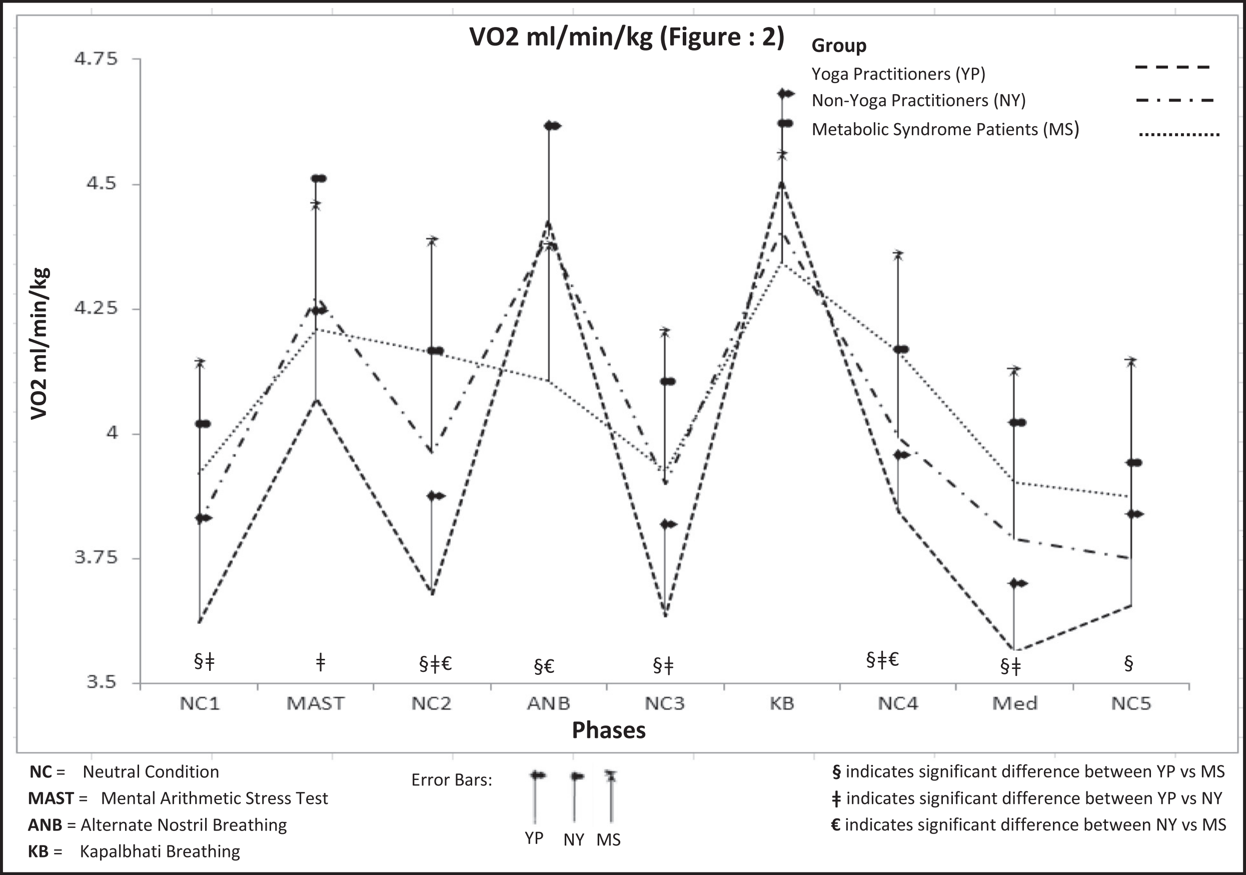
VO2 ml/min/kg (Figure : 2)
Group
Yoga Practitioners (YP)
Non-Yoga Practitioners (NY)
Metabolic Syndrome Patients (MS)
VO2 ml/min/kg
4.75
4.5
4.25
4
3.75
3.5
§‡
‡
§‡€
§€
§‡
§‡€
§‡
§
NC1
MAST
NC2
ANB
NC3
KB
NC4
Med
NC5
Phases
NC = Neutral Condition
MAST = Mental Arithmetic Stress Test
ANB = Alternate Nostril Breathing
KB = Kapalbhati Breathing
Error Bars:
YP
NY
MS
§ indicates significant difference between YP vs MS
‡ indicates significant difference between YP vs NY
€ indicates significant difference between NY vs MS
